# Prediction, isolation, overexpression and antifungal activity analysis of Medicago truncatula var. truncatula putative thaumatin like proteins (TLP-1, -2, -3, -4 and -5)

**DOI:** 10.3906/biy-1912-18

**Published:** 2020-08-19

**Authors:** Maryam SAEIDI, Reza ZAREIE

**Affiliations:** 1 Department of Biotechnology, Faculty of Agriculture, Isfahan University of Technology, Isfahan Iran

**Keywords:** Antifungal activity, *Medicago truncatula*, pathogenesis related proteins, protein expression, thaumatin like protein

## Abstract

Pathogenesis-related proteins (PR-proteins) are induced in response to environmental stresses such as osmotic and drought stress, wounding, microbial infections and treatment with specific plant hormones and elicitors. These proteins are classified into several groups (PR-1 through PR-17) based on their amino acid sequence and biochemical functions. The present study focuses on prediction, isolation, over-expression and analysis of the antifungal activities of the thaumatin-like proteins (i.e. PR-5) in the model legume *M. truncatula var. truncatula*. Analysis of *M. truncatula* genome sequence, available freely on the NCBI website, indicated the presence of at least 15 PR-5 Open Reading Frames (ORFs), 5 of them (dubbed TLP-1, -2, -3, -4 and -5) were selected for this study. Using gene-specific primers, the genomic coding sequences were isolated, sequenced and all confirmed to match with those reported in the database. All the fragments were, then, cloned in *Escherichia coli* isolate BL21 (DE3), using pET-21c(+) plasmids for subsequent overexpression (overexpression). All 5 genes were expressed as inclusion bodies (IBs) with masses, estimated by SDS PAGE, corresponding to the theoretical values. As expected, none of the protein IBs had no detectable effect on the phytopathogenic fungi *Rhizoctonia solani*,* Alternaria alternata*, *Fusarium graminearum*, *Fusarium solani*, *Verticillium *sp. and *Phytophtora *spp. However, when the in vitro refolded IB preparations were applied, all displayed comparable strong antifungal activities against the tested fungi. The current study is the first report of overexpression and evaluation of antifungal activities of PR-5 family of proteins from *M. truncatula* *Var. *t*runcatula*, and provides experimental evidence that all investigated proteins have the potential for enhancing resistance against some important fungal pathogens.

## 1. Introduction

Plants are in constant contact with microorganisms, many of which are potentially pathogenic, but the infection is a relatively rare event that usually involves species-specific pathogens. Plant disease resistance is elicited by specific recognition of pathogen-derived molecules. The recognition then leads to an array of resistance responses including the hypersensitive reaction (HR) at the site of pathogen entry and induction of the systemic acquired resistance (SAR) immune response. SAR provides protection not only at the site of infection but also in distant uninfected plant parts against a wide range of pathogens and is correlated with the expression of pathogenesis-related (PR) proteins, many with antimicrobial activities (Zhang et al., 2010). Pathogenesis-related proteins were first described based on their accumulation in tobacco leaves infected with Tobacco Mosaic Virus (TMV). A defensive role for these proteins in plant systems is suggested by their induction during the pathogen attack, and by their antifungal activity in vitro and in vivo. Genes encoding these proteins are now considered as an arsenal for the molecular breeding of pathogen-resistant plants. (Boccardo et al., 2019).

PR proteins are classified into 17 families (PR-1 to PR-17) (Van loon et al., 2006). Amongst which, PR-5 (or thaumatin-like) proteins are a group of highly soluble strongly antifungal proteins that originally detected as the slowest moving band among several low-molecular-weight proteins resolved by polyacrylamide gel electrophoresis analyses of acidic extracts of tobacco leaves challenged with TMV. They were related to the intensely sweet-tasting protein, thaumatin, from the West African shrub, *Thaumatococcus daniellii* based on sequence analysis (Van der Wel and Loeve, 1972). Other members of the thaumatin-like proteins (TLPs) are known as permatins (Skadsen et al., 2000) and osmotins (Anzlovar and Dermastia, 2003).

Mature TLPs fall into 2 size ranges, 1 group with a size range of 22 to 26 kDa (201 to 229 amino acids), and the other group with sizes around 16 kDa (148 to 151 amino acids) (Bryngelsson et al., 1989; Rebmann et al., 1991; Frendo et al., 1992; Piggott et al., 2004). TLPs have isoelectric point (pI) values ranging from very acidic to very basic (3.4 to 12). The proteins are generally resistant to proteases and pH-or heat-induced denaturation, characteristics partly due to the presence of 16 cysteines, shown to be involved in the formation of 8 disulphide bonds (Roberts and Selitrennikoff, 1990; Velazhahan et al., 1999; Selitrennikoff, 2001). TLPs are shown to be induced by various biotic and abiotic stresses such as microbial infection (Zhang et al., 2018), osmotic stress (Misra et al., 2016), wounding and application of abscisic acid (Frendo et al., 1992), ethylene, salicylate, methyl jasmonate as well as elicitors (Kitajima and Sato, 1999; Velazhahan et al., 1999).

In the present study, we predicted 5 TLP open reading frames from *M.truncatula Var. truncatula* using bioinformatic methods. Encoding sequences were isolated from *M. truncatula* genome, overexpressed in *E. coli* and the bioassay analysis was performed with the prepared proteins on some important agricultural fungi. This study was the first report on analysis of PR-5 family of PR-proteins from barrel medic.

## 2. Materials and methods

### 2.1. Materials

*M. truncatula* Gaertn cv Jemalong genotype A17 seeds were obtained from the south Australian Research and Development Institute SA, Australia. Restriction enzymes, *Pfu* and *Taq *DNA polymerases were purchased from Thermo Fermentas. All primers were manufactured by Metabion, Germany. pET-21c(+) expression vector and *E. coli *BL21 (DE3) strain were obtained from Novagen. Anti-Rabbit IgG Goat Polyclonal IgG-Peroxidase antibody and diaminobenzidine (DAB) were purchased from Sigma. Centricon centrifugal filter devices YM-10 were bought from Millipore. *R. solani*,* A. alternata*,* F. graminearum, F. solani*, *Verticillium *sp. and* Phytophtora *spp. were kindly provided by Dr B. Sharifnabi, Isfahan University of Technology. 

### 2.2. Bioinformatic analyses

The genome of *Medicago truncatula* was searched against well-characterized plant TLP sequences using the NCBI tblastn search algorithm1NCBI (2006). Blast Overview [online]. Website https://www.ncbi.nlm.nih.gov/genbank/ [accessed 1 January 2006].. Open reading frames were analysed by FGENESH2Softberry (2006). Annotation of Plant Genome [online]. Website http://linux1.softberry.com/berry.phtml/ [accessed 1 January 2006]., Geneseqer3PlantGDB (2019). Annotate a gene structure [online]. http://www.plantgdb.org/ [accessed 1 January 2019]., Genscan4GenScan (2009). Identification of complete gene structures in genomic DNA [online]. Website http://argonaute.mit.edu/GENSCAN.html [accessed 1 November, 2009]. , and Genemark5GeneMark (2019).GeneMark.hmm [online]. Website http://exon.gatech.edu/GeneMark/gmhmme.cgi [accessed 1 January 2019]. software. Signal peptides in the predicted proteins were analysed by the Expasy Signalp software6Improved prediction of signal peptides (2004). SignalP 3.0. [online]. Website http://www.cbs.dtu.dk/services/SignalP/ [accessed 1 January 2006].. Biochemical characteristics of putative mature proteins were calculated using the Expasy ProtParam7ExPASY (2006). ProtParam [online]. Website https://web.expasy.org/protparam/ [accessed 1 January 2006]. and Compute pI/Mw software8ExPASY (2006). Compute pI/Mw [online]. Website https://web.expasy.org/compute_pi/ [accessed 1 January 2006].. Pfam and SMART domain analysis programs were used with Expasy interproscan9http://www.ebi.ac.uk/Tools/InterProScan/ to predict conserved domains in the putative proteins. Sequence alignment was performed with EMBnet-CH/SIB Basic BLAST10ExPASY (2006). EMBnet [online]. Website https://embnet.vital-it.ch/ [accessed 1 January 2006 for protein/protein similarity in swiss-prot database and NCBI translated nucleotide database using a protein query blast (tblastn) against Reference mRNA sequences (Refseq rna) and nonhuman, nonmouse ESTs. This process was performed with MEGA_X_10.1.7 using the neighbour joining method and amino acid p-distance model (Kumar et al., 2018). Previously characterized TLPs were used as reference protein sequences.

### 2.3. Plant growth and DNA isolation 

*M. truncatula* seeds were germinated and grown for 21 days on MS medium (Murashige and Skoog, 1962) in a plant growth chamber adjusted to 25 °C, the light intensity of 2500 lux and a light/dark photoperiod of 16/8 h. Leaves were harvested, frozen in liquid nitrogen and subsequently kept at –20 °C until used. Genomic DNA isolation involves a modified cetyltrimethylammonium bromide (CTAB) procedure based on that of Murray and Thompson (1980) and additional extraction with chloroform and correct proportion of 2-mercaptoethanol.

### 2.4. PCR amplifications of TLP coding sequences

Polymerase chain reactions (PCRs) were performed using *Pfu* DNA polymerase following render instructions. The primers used to amplify fragments are shown in Table 1. DNA amplifications were performed in a thermal cycler using initial denaturation at 94 °C for 5.0 min, followed by 35 cycles of 1.0 min at 94 °C, 1.0 min at 52 °C, and 2.0 min at 72 °C. PCRs were terminated with a final extension step of 5.0 min at 72 °C. The products were resolved on a 0.7% w/v TAE agarose gel and visualized by ethidium bromide staining. TLP- 1, -2, -3 and -5 PCR products were purified from agarose gels using the Fermentas purification kit (K0513) and then prepared for T/A cloning by incubating 400 ng of the purified product with 2 mM dTTP, 0.2 mM BSA, 1 mM MgCl2, 0.13 unit *Taq* DNA polymerase and 1X *Taq* PCR buffer for 45 min at 70 °C. TLP- 4 PCR product was treated with *Nde*I and *Eco*RI enzymes to produce sticky fragments and then purified from 0.7% w/v TAE agarose gels.

**Table 1:** The sequences of designed primer.

Primer name	Sequence of primer (5’-3’)
F-tlp1	atgacaacattcacactcgttaacaaatgca
R-tlp1	tcaccttgggctgttgttagtggagg
F-tlp2	cctaacaggttcttatatgacaacattcacaatt
R-tlp2	gagctgatgtaatctgctacctgca
F-tlp3	atgctaatattcacctttgtaaaaactgcc
R-tlp3	cctcatcactttcaagtttcaatggc
F-tlp4	caagcatatgggaagattcaatatcaca
R-tlp4	atcagaattccatagatgtacaaaggaatcc
F-tlp5	atggcatcagtagtattttacaacaagtgtcc
R-tlp5	accaaacacagaataatatccatgacatcac

### 2.5. Construction of the expression plasmids

For TLP -1, -2, -3 and -5 pET-21c (+) was cut with *Nde*I and then treated with 2 mM dATP, 0.2 mM dTTP, 0.2 mM BSA, 1.25 mM MgCl2, 0.1-unit Taq DNA polymerase and 1X Taq PCR buffer for 45 min at 70 °C. These plasmids, with dA tails, were then ligated with coherent PCR products to produce the expression constructs which were subsequently transformed into competent *E. coli* K12 strain MC1061 cells. Directions of the ORFs in plasmids were analysed by a combination of the fragment-specific reverse primer and the T7 promoter primer (5’- taatacgactcactataggg -3’), this step results in >800 bp products in correctly positioned fragments whereas no such product was generated in reverse-positioned fragments. These analyses were confirmed with enzymatic digestion by *Mlu*I, *Pst*I, *Hin*dIII and again *Hin*dIII enzymes for TLP- 1, -2, -3 and -5, respectively. The amplified TLP- 4 PCR products were cloned into *Nde*I/*Eco*RI sites of pET-21c (+) vector and the constructs introduced into *E. coli* K12 strain MC1061 cells. Enzymatic digestion with *Pst*I was done to confirm the correction of TLP- 4 cloning. 

### 2.6. Expression of proteins

Expression plasmids, extracted from *E. coli *MC1061, were introduced into *E. coli *BL21 (DE3) strain. Single colonies containing the plasmids were grown in 100 mL TB medium containing 100 mg/mL ampicillin with vigorous shaking at 37 °C until an OD600 of 2.0–2.5 was obtained. Expression was induced by adding isopropyl β-D-thiogalactopyranoside (IPTG) to a final concentration of 0.5 mM and growing for an additional 4 h at 37 °C. The cells were centrifugated at 18514g for 10 min and resuspended in 10 mL lysis buffer (50 mM Tris pH 8.0, 8% sucrose, 5% Triton X-100, 50 mM EDTA, 0.1 mg/mL lysozyme). Suspended cells were briefly sonicated to break the bacterial cell wall. Soluble and insoluble proteins were separated by spinning at 4629 g for 30 min. Pellets were resuspended in TE buffer (10 mM Tris-HCl pH 7.8, 1 mM EDTA). 

### 2.7. Protein refolding

For protein refolding 100 µL resuspended inclusion bodies were dissolved in 900 µL denaturation buffer consisting of 6.6 M guanidine hydrochloride, 50 mM Tris pH 8.0, and 0.1 mM u1d60. Renaturation of recombinant proteins was conducted by gradual dilution of the dissolved proteins into 9 mL renaturation buffer (1 M urea, 50 mM Tris pH 8.0, 0.5 mM glutathione and 5.5% sucrose) for TLP- 1, -2, -4 and -5. For TLP-3, renaturation buffer contained 4 M urea, 50 mM Tris pH 8.0, 0.5 mM glutathione and 5.5% sucrose. To remove the guanidine hydrochloride and urea, samples were dialyzed against 10 mM phosphate buffer pH, 7.0 containing 5.5% sucrose for TLP -1,  -2, -4, -5, and 10 mM phosphate buffer pH, 5.0 containing 5.5% sucrose for TLP-3. Samples were concentrated using centricon centrifugal filter devices YM-10.

### 2.8. Western blot analysis

Antizeamatin antibody (a gift from Dr. C.P. Selitrennikoff) raised in rabbits was used in western blot analyses. Protein samples were electrophoresed on 12% SDS-polyacrylamide gels and transferred onto a nitrocellulose membrane in a wet transfer blot for 2 h in 100V. The membrane was blocked in 0.5% tween 20 in phosphate buffered saline (PBS) for 2 h and subsequently incubated with antizeamatin polyclonal antibody (1:500 dilution) for 12 h. After washing the membrane with 0.5% tween 20 in PBS, the blot was incubated with peroxidase secondary antibody (1:5000 dilutions) for 2 h and then washed 3 times in 0.5% tween 20 in PBS. The membrane was twice incubated in 50 mM sodium acetate pH 5.0 for 15 min. Bands were detected colorimetrically by immersing the filter in substrate solution containing 0.2 mL of 1 mg/mL diaminobenzidine (DAB), 10 mL 50 mM sodium acetate pH 5.0 and 30 µL 30% H2O2 for 15 min. The reaction was stopped with distilled water.

### 2.9. In vitro antifungal activity testing

Fungal species were grown on PDA plates at 22 °C for 3 days. In vitro effects of refolded proteins on *R. solani*, *F. graminearum*, *F. solani*, *Verticillium sp*. and *Phytophtora spp*. were analysed by incubating 2 μg of proteins with hyphae of mentioned fungi. These mixtures incubated for 48 h in 22 °C in light/dark photoperiod of 16 /8 h. Protein free buffer was used as negative control.

The germination inhibition effect of TLPs against *A.* *alternata* was investigated in spore germination assay (Xu Hu et al. 1997). Refolded protein (2 μg) was added to 6 × 106 spore suspension as the sample and protein free buffer as the negative control. The plates were incubated at 22 °C for 48 h (16/8 h in light/dark).

## 3. Results

### 3.1. Gene prediction

**Figure 1:**
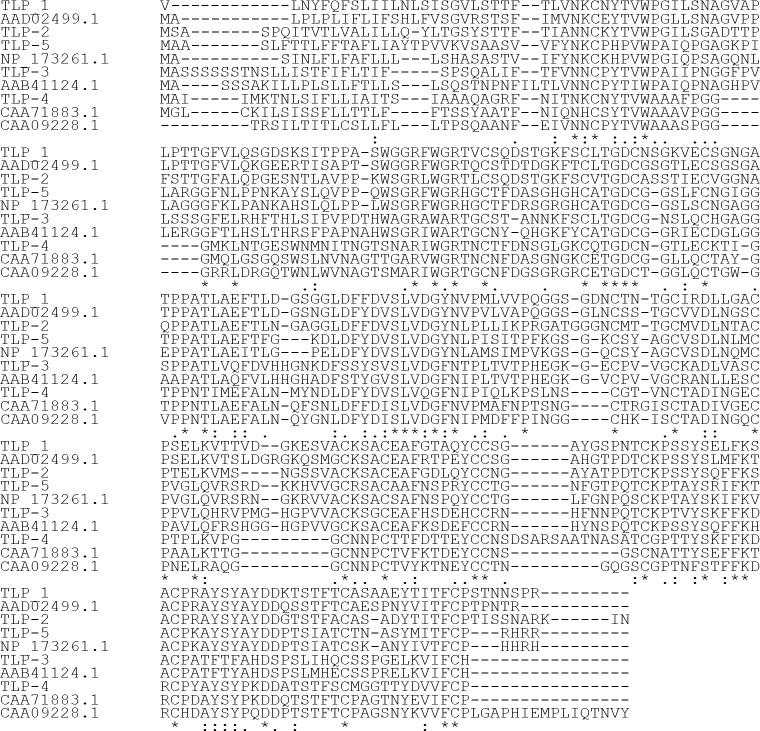
Alignment of amino acid sequences of TLPs with *A. thaliana* AAD02499, *A. thaliana* NP_173261, *V. vinifera* CAA71883, *C. arietinum* CAA09228, and *L. sculentum* AAB41124. Conserved residues among the aligned proteins are indicated as “*”.

**Figure 2:**
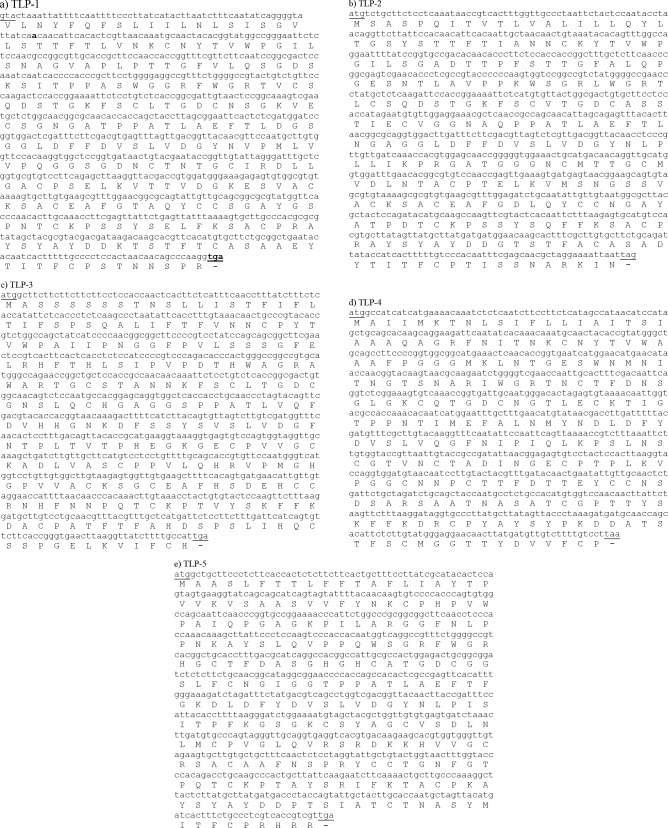
Nucleotide and deduced amino acid sequences of TLPs. The amino acid sequences are presented below the nucleotide sequences. Translation initiation and termination codons are underlined. Nucleotide and deduced amino acid sequences of signal peptides have been highlighted.

Plant thaumatin-like protein sequences including *Arabidopsis thaliana* TLPs (GenBank accession no. AAD02499, AAB71214 and NP_173261), *Lycopersicum sculentum* osmotin-like protein (AAB41124), *Vitis vinifera* osmotin-like protein (CAA71883), *Cicer arietinum* PR-5b (CAA09228) *Cryptomeria japonica* TLP (BAD90814), *Malus domestica *TLP (mald2) (Q9FSG7), *Prunus persica *TLP (AAM00215), and *Castanea sativa* TLP (CAB62167) were used as queries in tblastn searching of the *M. truncatula var. truncatula *genome. Analysis of barrel medic genome revealed the presence of at least 15 putative TLPs. Three of these putative proteins (TLP- 1, -2 and -5 belong to mth2-29h7, mth2-9i16, and mth2-10e12 *M.truncatula* *var. truncatula* clones, respectively) with 74% identities to *A. thaliana* AAD02499 for TLP-1, 63% again to *A. thaliana* AAD02499 for TLP-2, 78% to *A. thaliana* NP_173261 for TLP -5 were selected for this study. Two previously reported cDNAs (TLP- 3 and TLP- 4, with TC accession no. TC77106 and TC86847, respectively) were also selected for the present study. The expression of TLP- 4 mRNA was shown to be most strongly repressed by *Glomus mosseae* and *G. intraradices*, but TLP- 3 mRNA was shown to be induced by these fungi (Hohnjec et al., 2005). The highest identities of these proteins to well- known TLPs is 68% to *L. sculentum *AAB41124 for TLP- 3 and 56% homology to either *V. vinifera* CAA71883 or *C. arietinum* CAA09228 for TLP- 4. All 16 cysteine residues that are present in full length PR-5 proteins are conserved in TLP- 1, -2, -3, -4, and -5 (Figure 1). Expasy Signalp software predicted signal sequences at the amino- terminal suggesting that they are secreted proteins (Figure 2). Geneseqer, Genscan, Genemark, and FGENESH results revealed that none of these ORFs contain introns, although TLP- 5 appears to contain an intron in its signal peptide encoding sequence (Figure 2c), which was not important because these sequences were ignored in the primer design. Therefore, we cloned genomic DNA instead of cDNA for protein expression. Expasy protparam software calculated molecular mass of 24.0267 kDa and a pI of 4.8 for TLP-1, 24.2471 kDa and pI of 4.87 for TLP- 2, 24.1143 kDa and a PI of 6.98 for TLP- 3, 22.7203 kDa and a PI of 4.87 for TLP- 4 and 23.9953 kDa and pI of 9.0 for TLP- 5.

### 3.2. Expression of thaumatin-like proteins in E. coli

**Figure 3:**
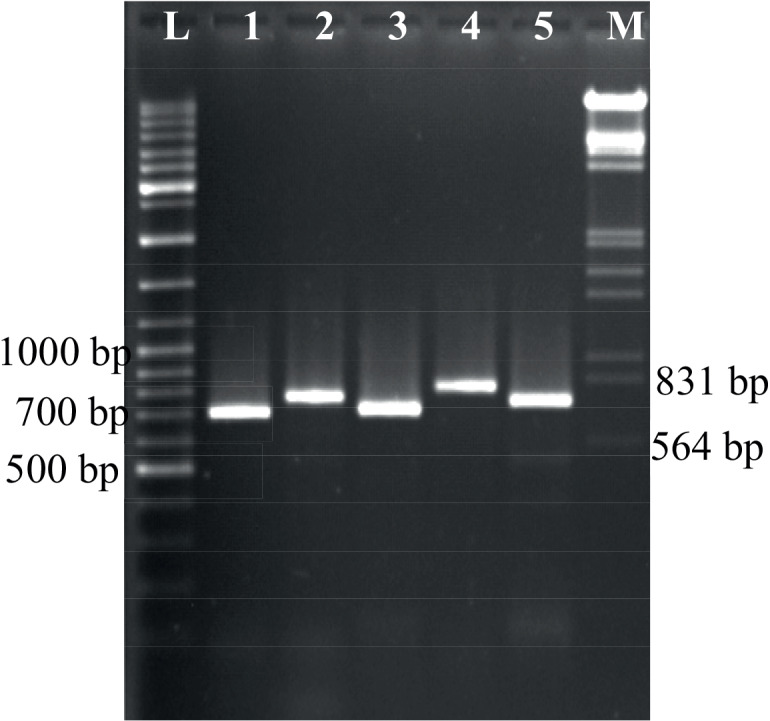
PR-5 amplicons separated in 0.7% TEA agarose gels. L: 100 bp plus ladder, 1: TLP-1 (699 bp), 2: TLP-2 (761 bp), 3: TLP-3 (696 bp), 4: TLP-4 (797 bp), 5: TLP-5 (735 bp), M: marker III

**Figure 4:**
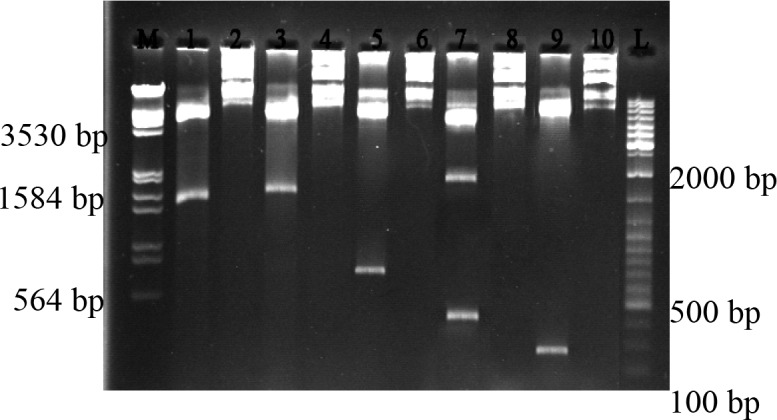
Enzymatic digestion of constructs having insert with correct orientation M: marker III, 1: cutted TLP-1, 2: uncutted TLP-1, 3: cutted TLP- 2, 4: uncutted TLP-2, 5: cutted TLP-3, 6: uncutted TLP-3, 7: cutted TLP-4, 8: uncutted TLP-4, 9: cutted TLP-5, 10: uncutted TLP-5, L: 100 bp plus ladder

**Figure 5:**
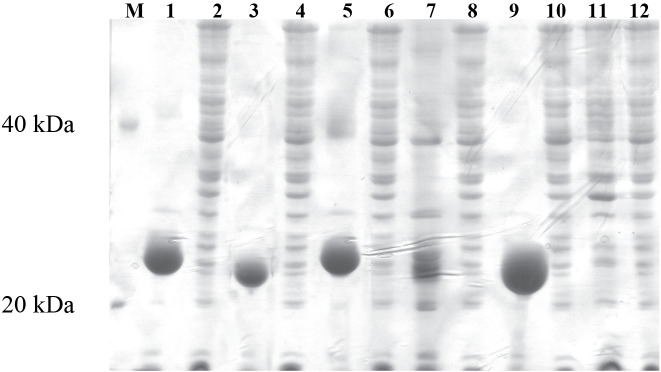
Protein expression M: protein marker, 1: expressed TLP-1 (IBs) (24.1579kDa), 2: nonexpressed TLP-1, 3: expressed TLP-2 (IBs) (24.3783 kDa), 4. nonexpressed TLP- 2, 5: expressed TLP-3 (IBs) (24.2455 kDa), 6: nonexpressed TLP-3, 7: expressed TLP-4 (IBs) (22.8515 kDa), 8: nonexpressed TLP-4, 9: expressed TLP-5 (IBs) (24.1265kDa), 10: nonexpressed TLP-5, 11: expressed BL21+ pET-21c(+), 12: nonexpressed BL21+ pET- 21c(+)

To study the antifungal activity of *M. truncatula* putative TLPs for the first time, we expressed and refolded TLP- 1, -2, -3, -4, and -5 ORFs to perform in vitro antifungal assays with the refolded proteins. We cloned 699, 761, 735, 789, and 696 bp fragments (*tlp*-1 to -5, respectively) into *E. coli *expression vector pET-21c(+) (Figure 3). To select the expression constructs having insert with the correct orientation, we used PCR techniques, enzymatic digestion (Figure 4) and sequence analysis (data not shown). The expressions were induced in the presence of 0.5 mM IPTG. SDS PAGE analysis revealed that TLP- 5, -3, and -1 were expressed in high amount, but TLP- 4 and -2 were expressed in low amount, respectively. Analysis of soluble and insoluble fractions from induced cultures has shown that the expressed recombinant proteins are present in the insoluble fraction as inclusion bodies (Figure 5). We analysed inclusion bodies activity against *A. alternata *spore germination, but no result was obtained. Therefore, we used different methods to solubilize the inclusion bodies to obtain the soluble form of proteins. We were able to solubilize all proteins in 6.6 M guanidine hydrochloride and removed denaturant to renature the protein using the procedure described in materials and methods. Although we used equal amount of recombinant proteins for refolding procedure but SDS PAGE analysis showed that recombinant proteins refolded in different percentages as: TLP- 5 > - 1 > - 2 > - 4 > - 3. 

To test if expressed proteins have any cross-reaction with other TLPs, these proteins were blotted and probed with antizeamatin antibody. All of them showed cross- reactivity with antizeamatin antibody.

### 3.3. In vitro antifungal activity

**Figure 6:**
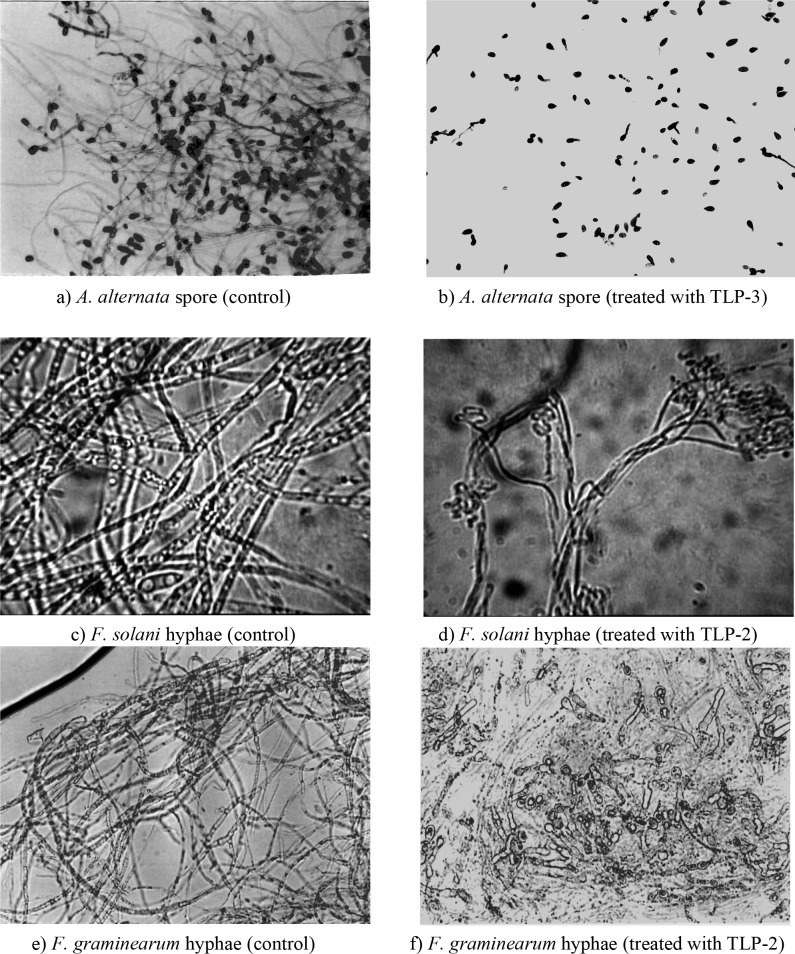

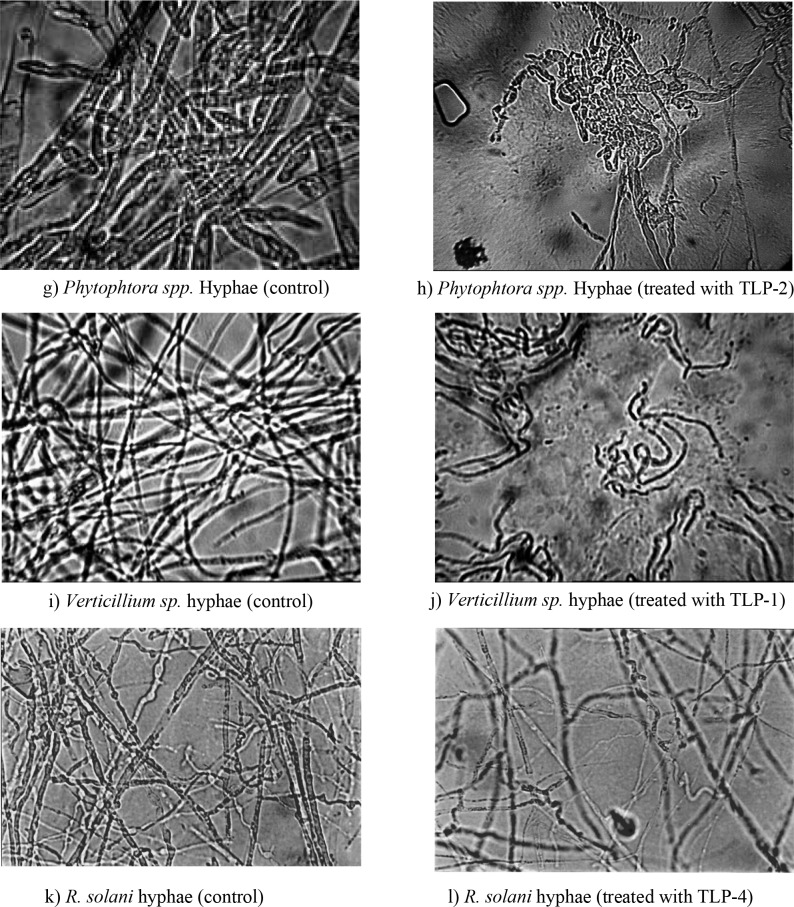
Bioassay results of TLPs on the tested fungi.

It was found that all TLPs reduced the viability of fungal hyphae. Spores of *A. alternata* showed significantly reduced germination in the presence of the proteins (Figure 6-a and b). β-1, 3-Glucan is the main structural component of the cell walls of this fungus. It is probable that *Medicago* TLPs function as β-1, 3-Glucanase-like proteins and weakened fungal cell walls to prevent spore germination. β-1, 3-Glucanase-like proteins bind to β-1, 3-glucan present in the cell surface of bacteria and fungi, triggering the activation of prophenol oxidase cascade, result in the formation of cytotoxic and antimicrobial compounds and thus represent an important defence mechanism in a variety of invertebrates (Grenier et al., 1999). Our assay revealed that TLP- 5, -4, and -3 have stronger inhibitory effect on *A. alternata* spore germination than TLP- 2 and TLP-1. Microscopic analysis of the hyphae indicated that TLPs may cause hyphal morphology modification of *F. graminearum* and *F.solani *(Figure 6c to f), *Phytophtora *spp. and *Verticillium *sp. (Figure 6g to j), prevention of hyphal branches for *R. solani* (Figure 6k and l). According to the present study, all TLPs caused hyphal malformation on *F.solani* and *F. graminearum*, *phytophtora *spp*. *(unless TLP- 3) and on *Verticillium *sp. (unless TLP- 5). To determine if Fusarium hyphae were damaged, some of the treated hyphae were placed on PDA medium and studied after 3 and 7 days of incubation at room temperature. The results showed that the TL protein-treated samples had less colony production than control on days 3 and 7. In summary, it seems that the studied proteins are capable of damaging the growth potential of Fusarium or at least significantly delaying its growth. Our assay also revealed that all proteins prevent the formation of hyphal branches on *R.solani*, the results of hyphal culture on PDA medium confirmed this. 

## 4. Discussion

In the present study, 5 new TLP encoding sequences from *M. truncatula* *var. truncatula* genome were isolated, overexpressed in *E. coli* as proteins that were tested for their antifungal activity. Three of the TLPs were acidic (TLP- 1, -2, and -4), 1 was basic (TLP- 5) and the last 1 was neutral (TLP- 3). The data presented here show that these proteins have structural and functional characteristics of TLPs. The amino acid sequences of these proteins have very high sequence identities with well-known TLPs and contained all 16 conserved cysteine residues, like some other TLPs. This structure is able to stabilize protein, and allows the protein to withstand proteases, pH, and heat-induced denaturation (Ghosh and Chakrabarti, 2008). The presence of hydrophobic N-terminal sequences (signal peptide) in the deduced amino acid sequences of these proteins suggests that TLPs are secreted proteins. Western-blot analysis results showed cross-reactivity of these proteins with zeamatin, a member of TLP group. Based on these results we reported them in NCBI as *M. truncatula* *var. truncatula *TLPs with accession number KM220900, KM220902, AM943532, KM220901, KM220903 for TLP-1 to -5, respectively.

It was reported that transgenic tobacco plants overexpressing rice and cotton fibre TLPs showed enhanced resistance to *A. alternate* (Velazhahan and Muthukrishnan, 2003) and *Verticillium dahliae* (Munis et al., 2010), respectively. Similarly, the antifungal activity of AdTLP in tobacco transgenic plants was reported against a wide range of plant pathogenic fungi such as *R. solani*, *F. oxysporum*, and *F. solani* (Singh et al., 2013).  In another study, TLP gene of *Camellia sinensis* (CsTLP) was overexpressed in transgenic potato plants to investigate the potential role of CsTLP in imparting tolerance to *Phytophthora infestans*. The leaves of CsTLP transgenic lines showed resistance to *P. infestans* spores under in vitro conditions (Acharya et al., 2013). 

Recombinant proteins were produced as inclusion bodies. These structures of proteins are composed of aggregates of unfolded, partially folded and misfolded protein and failing to reach a correct conformation in the cytoplasm. The protein found in inclusion bodies cannot be directly used for studies due to a lack of biological activity. Consequently, several refolding methods have been extensively reported. We have tested a generic denaturation/refolding protein purification procedure to assess refolded proteins. Using dialysis against a single buffer allowed us to obtain more soluble proteins. According to Karen et al., (2003) this protocol is an efficient way to generate structural samples for high-throughput studies of proteins. Since proteins are diverse in structure-functional properties, additives that work well for a protein may not function for another. The efficiency of refolding processes is affected by the nature, size, solubility of proteins, environmental conditions (ionic strength, pH, salt type, and salt concentration), and temperature (Moosavi-Movahedi et al., 2016). Medicago TLPs (TLP-1 to -5) also refolded in different amounts and we assume these factors affected the efficiency of protein refolding in present study. 

Growth inhibitory activities of the refolded proteins on some important fungi were also investigated via microscopic examinations. All proteins have displayed strong antifungal activities against *R. solani*,* A. alternata*,* F. graminearum*,* F. solani*,* Verticillium *sp. and* Phytophtora *spp. Overall, the findings suggest that TLPs are an effective member of TLPs with antifungal activity and presumably participate in plant defences as antifungal factors. 

The antifungal effect of TLPs is due to the inhibition of hyphal growth (Anand et al., 2004), spore lysis and/or reduction in spore germination or viability of germinated spores (Grenier et al., 1993). Aggregation of compact hyphal branches leads to infection peg formation which allows the pathogen to penetrate and proliferate further (Dodman et al., 1968). According to Zareie et al. (2002) it seems that TLPs degraded structural components of fungi. However, the mechanisms by which TLPs bring about these effects are not completely understood. Future studies will presumably reveal some more detail about their mechanism of action.
